# Among the Colleges

**Published:** 1897-10

**Authors:** 


### AMONG THE COLLEGES.

### UNIVERSITY OF PENNSYLVANIA.

Dr. John Marshall has been succeeded by Dr. Leonard Pearson as dean of the Veterinary Faculty. Dr. Herman A. Christman has succeeded Dr. C. E. Fouse as assistant demonstrator of veterinary anatomy. Dr. Milton E. Conard, in addition to being demonstrator of veterinary obstetrics, will also demonstrate veterinary surgery, as Dr. A. Salinger, who occupied this position, has been dropped from the list. Dr. Clarence J. Marshall will occupy the position of demonstrator of the theory and practice of veterinary medicine. Harrison K. Caner has been added to the Board of Managers. Dr. William G. Shaw is at present house-surgeon. A course in bacteriology is now required from second- and third-year students.

---

### CHICAGO VETERINARY COLLEGE.

Dr. James Henderson has succeeded Dr. M. R. Trumbower as professor of cattle pathology and milk- and meat-inspection, and Dr. S. E. Sayre has been succeeded by Dr. A. E. Flowers as professor of dental surgery. Dr. A. S. Nesbitt is assistant to the chair of theory and practice in place of Dr. F. R. Davis, of last year. With this session the college institutes a post-graduate course. Dr. Glass' translation of Dr. Müller's work on *Diseases of the Dog* has been added to the college's list of text-books.

---

A horseshoe, comprising an India-rubber block forming the body and recessed in the centre to leave the sole of the horse's hoof exposed, a protective metal band placed round the outer edges of the block, the metal band having projecting parts embedded in the rubber block, and a membrane over the recess, which is perforated to allow egress and ingress of air, is a recent invention.

